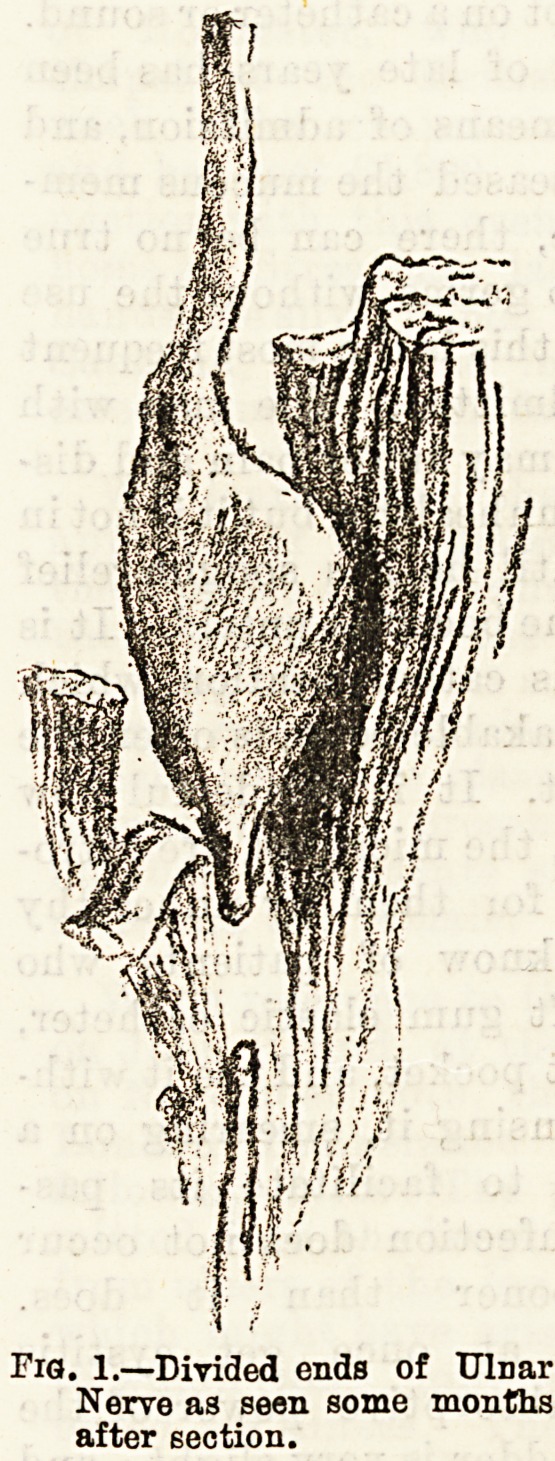# Traumatic Ulnar Nerve Paralysis.—II

**Published:** 1894-12-08

**Authors:** W. McAdam Eccles

**Affiliations:** Assistant Surgeon West London Hospital and City of London Truss Society, Assistant Demonstrator of Anatomy St. Bartholomew's Hospital, Surgeon St. Marylebone General Dispensary


### TRAUMATIC ULNAR NERVE PARALYSIS.—II.

By W. McAdam Eccles, M.B., B.S.Lond., F.R.C.S. Eng., Assistant Surgeon West London Hospital and City of London Truss Society, Assistant Demonstrator of Anatomy St. Bartholomew's Hospital, Surgeon St. Marylebone General Dispensary.

(*Continued from page* 153.)

As was shown in the first paper, traumatic ulnar nerve paralysis leads to very distressing results, and may, in the case of a mechanic, cause great misery from the loss of the functions of the hand. It is now an established fact, although long doubted, that nervous tissue can become regenerated, but only if the divided ends are brought accurately in contact with one another.

The treatment of a divided nerve may be discussed under the two headings—(1) primary suture, (2) secondary suture.

A man receives a somewhat deep cut across the wrist; it is of the utmost importance to one's patient that we determine whether or not he has divided tendons, vessels, or nerves. If a tendon has been cut through, it is the proper and usual treatment to at once suture it; if vessels have been wounded, it is necessary to ligature; but when we diagnose a lesion of a nerve trunk, what line of treatment should we adopt? An aseptic suture, preferably either of silk or fine kangaroo tail, inserted into nervous tissue will do no harm. This being so, it is obvious that to suture a nerve is the best method of bringing its separated ends in contact with one another. There need be no fear under the strict antiseptic, or better aseptic, treatment, of tetanus, neuritis, or other evils following on nerve suture. If a nerve which has been divided in a wound is at once sought for, and its ends carefully brought together by sutures passing right through the nerve substance, eminently satisfactory results will in the majority of cases follow. A nerve will practically never reunite unless sutured, or if it does it will only very imperfectly. When the apposition has been brought about, it is needful that the part be placed in such a position that the least possible strain may result, and having been put thus, it should be kept there by a splint or otherwise. Thus the wrist should in all cases be flexed where the nerve lesion, ulnar or median, is near it; but if the ulnar be divided behind the internal condyle, an extended position of the elbow is obviously the best. If healing by primary union occurs, the restoration of function will in most cases be rapid and complete.

Fig. 1.—Divided ends of Ulnar Nerve as seen some months after section.

If failure follows, or the nerve was not sutured at the time of the accident, the trophic changes which have been described will inevitably follow. A very interesting condition of the proximal end occurs. It becomes bulbous, the "bulb" being composed of a mass of fibrous tissue, in which are some newly-formed nerve fibres. (See Fig. 1.)

In cases of secondary nerve suture, it is necessary to refresh the ends of the nerve after they are found by making a longitudinal incision over the course of the nerve. It is not requisite to cut away all the bulbous end; indeed, the upper part of the bulb, where there are numerous young nerve fibres, is also tough, and affords an excellent hold for sutures. If the divided ends cannot be brought together, a piece of nerve from an amputated limb may be transplanted into the gap and fixed by sutures. It is usually well in these cases to render the operation bloodless, by applying an Esmarch's bandage, but this should be placed around the limb sufficiently high up in order that its pressure may not interfere with the approximation of the ends.

As to the length of time after division of a nerve that it is advisable to attempt reunion, there is good hope of success, or at any rate improvement even up to twelve months. As no harm can be done it is always advisable to try suture in the hope that some benefit may result. It is almost needless to repeat that asepsis is absolutely necessary for a good result in all these cases.

Pressure upon a nerve, as, for instance, upon the musculo-spiral in a case of fracture of the shaft of the humerus and the subsequent implication of the nerve in callus, always calls for treatment. At first it is usually only needful to see that degeneration of the muscles does not occur by the employment of galvanism and massage. The ulnar nerve, if involved in callus by a fracture of the internal condyle, will have all the symptoms described in the first paper. If after a reasonable time, say, two or three months, no improvement in the condition has become evident, it is the best treatment to cut down on the nerve and attempt to free it from the surrounding callus. Great care must in these cases be exercised in order not to divide the nerve itself while separating the surrounding tissues.

## Figures and Tables

**Fig. 1. f1:**